# Exploring Heat-Response Mechanisms of MicroRNAs Based on Microarray Data of Rice Post-meiosis Panicle

**DOI:** 10.1155/2020/7582612

**Published:** 2020-09-17

**Authors:** Yan Peng, Xianwen Zhang, Yuewu Liu, Xinbo Chen

**Affiliations:** ^1^Key Laboratory for Crop Gene Engineering of Hunan Province, Hunan Agricultural University, Changsha 410128, China; ^2^College of Information and Intelligence, Hunan Agricultural University, Changsha 410128, China

## Abstract

To explore heat response mechanisms of mircoRNAs (miRNAs) in rice post-meiosis panicle, microarray analysis was performed on RNA isolated from rice post-meiosis panicles which were treated at 40°C for 0 min, 10 min, 20 min, 60 min, and 2 h. By integrating paired differentially expressed (DE) miRNAs and mRNA expression profiles, we found that the expression levels of 29 DE-miRNA families were negatively correlated to their 178 DE-target genes. Further analysis showed that the majority of miRNAs in 29 DE-miRNA families resisted the heat stress by downregulating their target genes and a time lag existed between expression of miRNAs and their target genes. Then, GO-Slim classification and functional identification of these 178 target genes showed that (1) miRNAs were mainly involved in a series of basic biological processes even under heat conditions; (2) some miRNAs might play important roles in the heat resistance (such as osa-miR164, osa-miR166, osa-miR169, osa-miR319, osa-miR390, osa-miR395, and osa-miR399); (3) osa-miR172 might play important roles in protecting the rice panicle under the heat stress, but osa-miR437, osa-miR418, osa-miR164, miR156, and miR529 might negatively affect rice fertility and panicle flower; and (4) osa-miR414 might inhibit the flowering gene expression by downregulation of LOC_Os 05g51830 to delay the heading of rice. Finally, a heat-induced miRNA-PPI (protein-protein interaction) network was constructed, and three miRNA coregulatory modules were discovered.

## 1. Introduction

As the staple food, rice (*Oryza sativa*) is consumed by more than half the world population. Heat stress is one of the critical factors in limiting plant growth and productivity [1, 2]. According to the International Rice Research Institute (IRRI), the yield declines by 10% for each 1°C increase during the sensitive period of rice growth and in the sensitive temperature range [3]. Therefore, understanding the molecular basis of rice in response to the heat stress is necessary and important to sustain the rice production.

Plant miRNAs are a kind of endogenous noncoding small RNAs, which regulate the gene expression by the mRNA degradation or translation inhibition [4]. In recent years, miRNAs have been reported as key players in plant tolerance to the heat stress [5]. For example, the expression of miR397b.2 was induced by heat stress, and its target gene L-ascorbate oxidase was downregulated under the heat stress [6]. Under the heat stress, miR160, miR169, and miR1884 showed differential expression in shoot and root tissues for rice cultivar Nagina 22 [7]. By using the next-generation sequencing (NGS) and degradome sequencing (DS), ten miRNAs with significant expression changes were related to the heat stress. They were composed of seven upregulated miRNAs, including miR160e, miR160f, miR396a, miR396e, miR164a, miR166k, and miR166g, and three significantly downregulated miRNAs, such as miR5054, miR408, and miR5788 [8]. The negative correlation between miR169r-5p and its target gene LOC_Os12g42400 was confirmed under the heat stress, and the overexpression of miR169r-5p in rice at the flowering stage resulted in enhanced heat tolerance [9]. Li et al. concluded that 21 miRNAs were upregulated and 26 miRNAs were downregulated under the heat stress. Among them, miR162b, miR171b, miR169n, miR529a-p5, and PC-5P-62245-9 were verified by the quantitative real-time PCR [10]. However, heat response mechanisms of miRNAs in the rice post-meiosis panicle are still not well understood.

**Figure 1:**
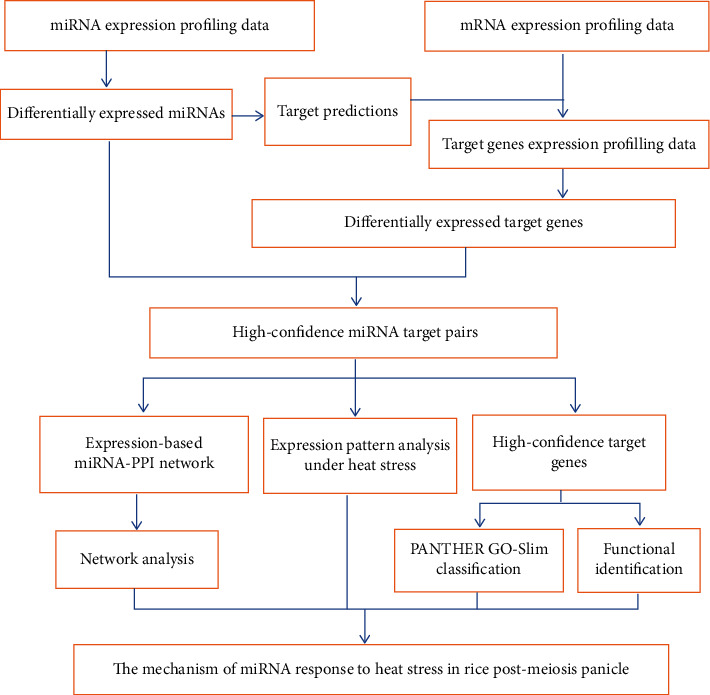
Flowchart of the general procedure of this study.

In this work, we showed the time course for the miRNA expression profile in the post-meiosis panicle of rice cultivar 996 treated at 40°C from 0 min to 2 h using the Agilent 4x44K rice microarray to identify heat-responsive miRNAs in the rice panicle. Then, high-confidence miRNA target pairs were identified. Furthermore, based on the function analysis and miRNA-PPI network construction, three miRNA coregulatory modules were discovered and heat-responsive mechanisms in the rice post-meiosis panicle were studied. The schematic diagram of the procedures is given in Figure 1.

## 2. Material and Methods

### 2.1. Plant Materials and Heat Stress Treatments

Rice cultivar 996 was cultivated in a ten-square metre experimental rice field at Hunan Agricultural University under the same growth conditions. For the heat treatment, we moved six rice plants into the growth chamber (Binder, Tuttlingen, Germany) when they reached anther development stage 10 of post-meiosis defined by Zhang and Wilson [11]. These plants were pretreated in the growth chamber under the condition of 32°C/28°C (day/night) with 80% humidity for one day (12 h/12 h), and then the heat treatments were done at 40°C with 80% relative humidity and illumination intensity of 600 *μ*mol m^−2^ s^−1^ for 0 min (used as control), 10 min, 20 min, 60 min, and 2 h. Young florets at the anther development stage 10 at the middle of the main panicles were harvested into the liquid nitrogen immediately at these five time points and stored in −80°C for further usage.

### 2.2. RNA Isolation, Microarray Hybridization, Signal Scanning, and Normalization

Microarrays were performed on RNA isolated from samples by using TRIzol (Invitrogen, Carlsbad, CA) and further purified using a QIAGEN RNeasy kit (Qiagen, Valencia, CA). Two independent samples were assayed at each time point for the experimental groups. Gene expression profile analysis was performed by using the Agilent 4x44K rice oligo microarray with 42,489 60-meroligonucleotide probes. Ten microarrays were performed by using the single dye defaults for all parameters.

These above hybridization signal data were obtained by using the Agilent Feature Extraction software, and then the data files were imported into GeneSpring GX (Agilent Technologies). We used specific flags of P, A, and M to mark the detected, not detected, and compromised data, respectively. These data were normalized by quantile algorithm followed by the process of baseline to median of all samples.

These original microarray data were submitted to the Gene Expression Omnibus and assigned the accession number GSE51426.

### 2.3. Data Filtering

Data filtering was performed with the criteria of at least 6 out of 10 samples with the P flag to ensure the quality of the normalized data. To identify heat-responsive miRNAs and genes, GEO2R analysis was applied to identify differentially expressed miRNAs and genes between the heat stress sample and the control sample. The criteria to consider a miRNA or a gene to be differentially expressed was ∣log2 fold change | ≧1.0 and *P* value < 0.05 for at least one time point.

### 2.4. Replicate Analysis and Quantitative Real-Time PCR (qRT-PCR) Verification

We have previously reported the replicate analysis and qRT-PCR verification on microarray data [12]. The results in it showed that there were good correlations between biological replicates, and the qRT-PCR expression of all candidate genes was consistent with microarray data.

### 2.5. Target Prediction of miRNAs and High-Confidence miRNA Target Gene Identification

Target prediction was performed by using the plant miRNA target analysis online software psRNATarget [13]. The default parameters were maintained except the expectation value (set as 3) to minimize the number of nonauthentic targets. MSU Rice Genome Annotation for Version 7 was selected as the cDNA library.

After we got the differentially expressed miRNA (DE-miRNA) putative target genes, we compared them to the normalized data and got the expression profile data of putative target genes under the heat stress in the rice post-meiosis panicle. Then, we compared the expression patterns of DE-miRNA families with their differentially expressed target genes (DEGs). The expression levels of DE-miRNA families and their targets, which altered in the opposite direction within the any time period, were considered as high-confidence miRNA target genes.

### 2.6. Functional Identification of High-Confidence miRNA Target Genes

To understand the functions of DE-miRNAs, we carried out functional identification of their high-confidence target genes. The GO-Slim classification was performed based on the GO database and powered by the PANTHER Classification System (http://www.pantherdb.org/). The OGRO database (http://qtaro.abr.affrc.go.jp/ogro) [14] was employed to identify the functionally characterized genes.

### 2.7. Network Visualization and Construction

The protein-protein interaction (PPI) of the high-confidence target genes was analyzed by using STRING 11 (https://string-db.org/cgi/input.pl). The PPI with a combined score (1: highest confidence; 0: lowest confidence), which was larger than 0.7, was used for the next step network analysis. Cytoscape_v3.7.2 was utilized to construct the interaction network of miRNA-PPI and the bipartite network of DE-miRNA families under the heat stress in rice post-meiosis panicles.

## 3. Results and Discussions

The normalized data of expressed miRNAs and genes at the time points of 0 min, 10 min, 20 min, 60 min, and 2 h after heat treatment are listed in Supplementary Table S1. MicroRNA-mature data were used for further differentially expressed analysis.

### 3.1. Differentially Expressed miRNAs during the Heat Treatment

**Figure 2:**
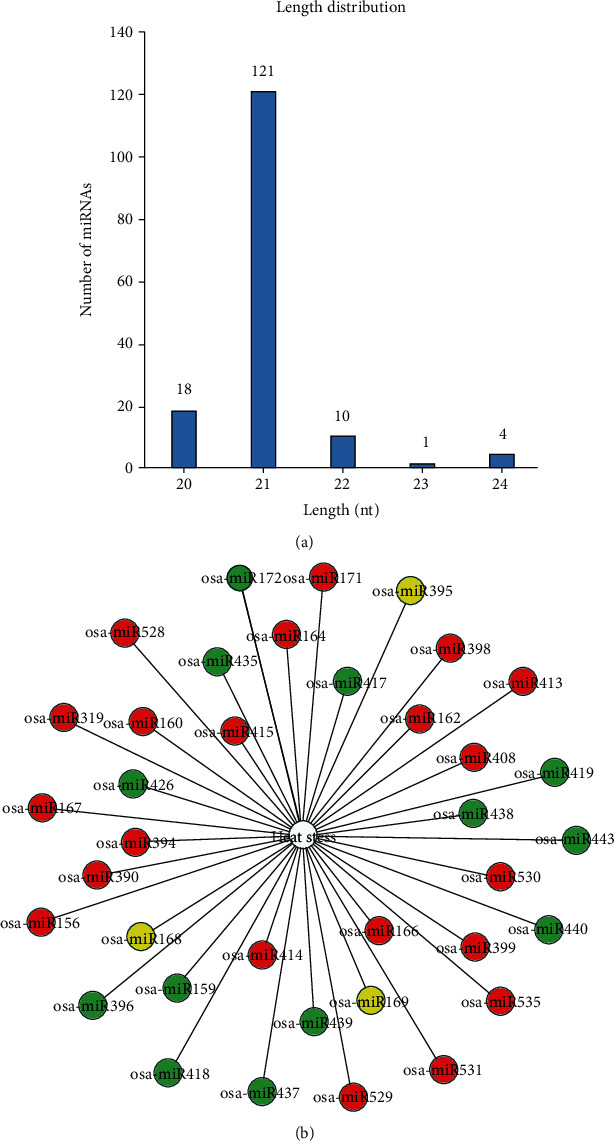
Classification of DE-miRNAs in rice under the heat stress. (a) The length distribution of 124 DE-miRNA sequences. (b) Bipartite network visualization of the regulatory relationship between DE-miRNA families and heat stress in rice. The links between heat stress and miRNA families indicate involvements between miRNA families in the regulation of heat response. The solid red, green, and yellow circles denote upregulated, downregulated, and up-/downregulated expression patterns of miRNA families under the heat stress, respectively.

A total of 124 miRNAs were identified to be differentially expressed in rice under the heat stress, corresponding to 37 rice miRNA families. By mapping 124 DE-miRNAs to the miRBase database (release22.1; http://www.mirbase.org/), we obtained 154 mature sequences. The list of 124 DE-miRNAs and their mature sequences are provided in Supplementary Table S2. Statistical analysis showed that 86 of 124 DE-miRNAs were upregulated, and 38 of them were downregulated. The length distribution of the DE-miRNA sequence showed that most of them were 21 nt (Figure 2(a)). Bipartite network visualization of the regulatory relationship between DE-miRNA families and heat stress in rice is shown in Figure 2(b). It showed that 21 of 37 DE-miRNA families were upregulated, 16 of them were downregulated, and 3 of them were up-/downregulated (either upregulated or downregulated). The expression patterns of miRNAs in the same family were consistent under the heat stress except for osa-miR168, osa-miR169, and osa-miR395. In these three miRNA families, only 6 miRNAs were downregulated, which were osa-miR168a, osa-miR169g/n/o, and osa-miR395c/o, and the other 23 miRNAs were upregulated. Our results indicated that the majority of DE-miRNAs and DE-miRNA families were upregulated under the heat stress.

A closely related report [10] showed that 47 miRNAs were identified to be differentially expressed in young panicles in rice cultivar N22 by using high-throughput deep sequencing. Among them, 26 miRNAs were downregulated and 21 miRNAs were upregulated. The numbers of upregulated miRNAs and downregulated miRNAs were balanced in rice young panicles. However, in our study, the number of upregulated miRNAs was much more than the number of downregulated miRNAs. Under the heat stress, osa-miR159a, osa-miR162b, osa-miR160a/f, osa-miR166i/h, osa-miR159a/f, osa-miR169n, osa-miR390, and osa-miR398b had the same expression pattern among the two cultivars. However, the expression of osa-miR529, osa-miR399d, osa-miR156d, osa-miR169h, and osa-miR167h was specific to varieties.

### 3.2. Target Prediction and High-Confidence miRNA Target Gene Discovery

The biological functions of miRNAs are known to be intimately relevant to the functions of their target genes. Therefore, identification of the potential target genes can provide an effective approach to study the complex miRNA-mediated regulatory mechanisms. In this work, a total of 1289 putative target genes (Table S3-1) were predicted through prediction and removing repeated genes. Then, we compared 1289 putative target genes with data in Supplementary Table S1. A total of 838 putative target genes were obtained under the heat stress in rice post-meiosis panicles (Table S3-2). Finally, 351 putative target genes (41.89%) were identified to be differentially expressed (Table S3-3).

In order to find high-confidence miRNA target genes, we compared the expression patterns of 37 DE-miRNA families with their 351 DEGs. The results showed that the expression patterns of 29 DE-miRNA families were completely opposite to that of 178 DEGs (Table 1). These 178 DEGs might be high-confidence target genes for their miRNAs. 178 high-confidence miRNA target gene descriptions are provided in Supplementary Table S4. These target genes consisted of transcription factors, hydrolase, kinases, zinc finger, disease resistance protein, nucleic acid binding proteins, etc., and most of them were downregulated to a different extent under the heat stress. High-confidence target genes were distributed on every chromosome. However, chromosomes 1, 2, and 3 of rice had higher frequencies of targets.

Previous studies confirmed the reliability of the following high-confidence miRNA target pairs, including osa-miR156-*OsSPL7* [15], miR529-*SPL7* [16], miR172-*SHAT1* [17], miR164-*OMTN4* [18], miR168-*AGO1* [19], and miR159-*GAMYBL2* [20]. LOC_Os02g53620, LOC_Os03g07880, LOC_Os03g44540, and LOC_Os12g42400, as high-confidence target genes of osa-miR169, were members of the NF-YA family genes. Li et al. asserted that miR169/NF-YA modules regulated tolerance to abiotic stresses in both monocots and dicots [21]. LOC_Os02g47280, LOC_Os02g53690, LOC_Os06g02560, and LOC_Os12g29980, as high-confidence target genes of osa-miR396, were members of the Growth-Regulating Factor (GRF). Liu et al. concluded that miR396 could target six GRF genes, encoding putative transcription factors with roles in the plant leaf growth [22]. LOC_Os02g43660 and LOC_Os06g11490 were uclacyanin (UCL) genes of the phytocyanin family. osa-miR408 regulated the grain yield by downregulating its downstream target OsUCL8, which was an UCL gene of the phytocyanin family [23]. All these information indicated that high-confidence miRNA target pairs had certain reliability. These high-confidence miRNA target genes provided candidates for elucidating the mechanism of miRNA involvement in the heat stress tolerance in rice post-meiosis panicles.

### 3.3. Expression Patterns of High-Confidence miRNA Target Pairs

**Figure 3:**
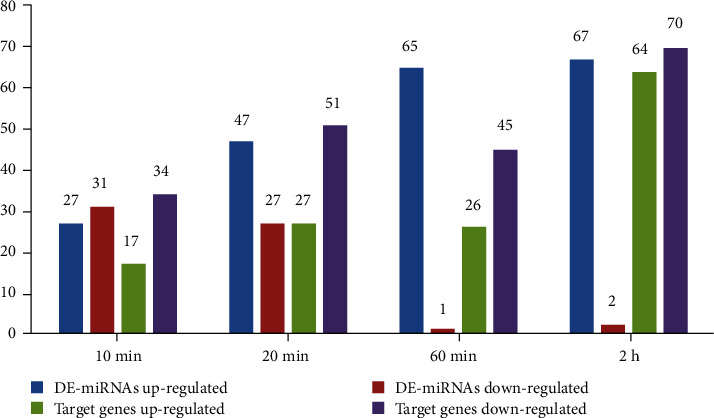
Numbers of high-confidence target genes and their DE-miRNAs at the time points of 10 min, 20 min, 60 min, and 2 h after the heat treatment. The horizontal axis represents the time after the heat treatment, and the vertical axis represents the number of up- and downregulated probes.

The expression patterns of 178 high-confidence target genes and their DE-miRNAs at the time points of 10 min, 20 min, 60 min, and 2 h after the heat treatment are shown in Figure 3. As was shown in Table 1, a miRNA was matched to a few target genes. The number of upregulated DE-miRNAs increased with the time and remained at a high level. However, the number of downregulated DE-miRNAs decreased with the time and remained at a lower level at 60 min and 2 h. Figure 3 shows that both upregulated and downregulated high-confidence target genes were increasing with the time, reaching the highest level at 2 h, and the number of downregulated genes was significantly higher than that of the upregulated genes. These results showed that most miRNAs resisted the heat stress by downregulating their target genes. In addition, the number of downregulated DE-miRNAs after the heat treatment for 10 minutes was significantly higher than that of upregulated target genes, while the number of downregulated DE-miRNAs after the heat treatment for 60 min and 2 hours was significantly lower than that of upregulated target genes. These results indicated that there might be a time lag between miRNA expression and their target gene expression.

### 3.4. PANTHER GO-Slim Classification

**Figure 4:**
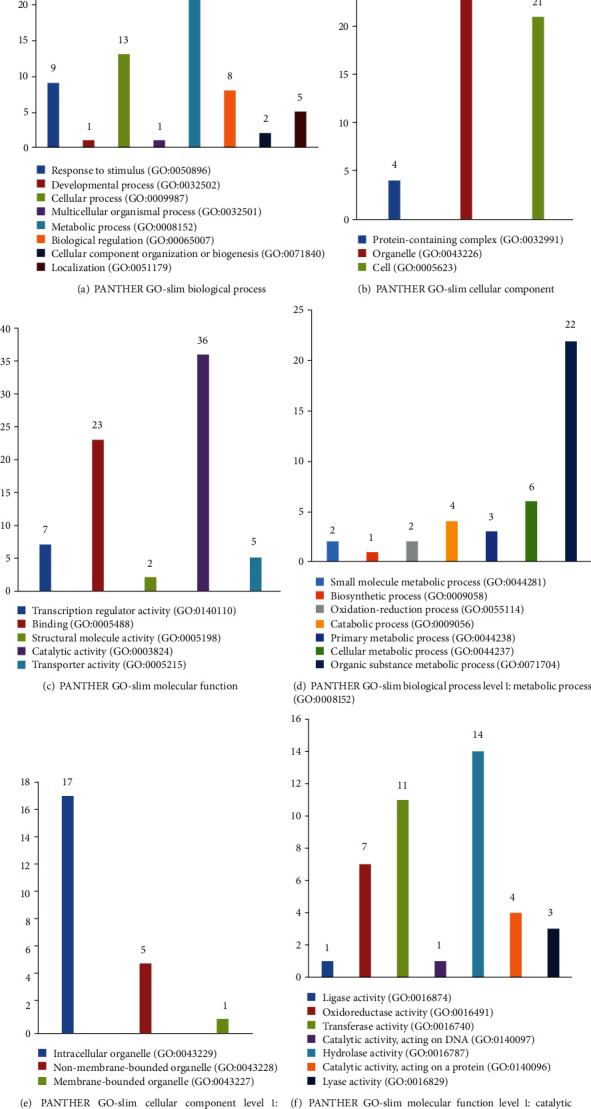
PANTHER GO-Slim classification of high-confidence target genes under the heat stress. The vertical axis represents the number of high-confidence target genes.

178 high-confidence target genes were mapped to the PANTHER Classification System to explore their biological significance. According to the PANTHER GO-Slim classification, target genes belonged to eight important biological processes (BP): response to stimulus, developmental process, cellular process, multicellular organismal process, metabolic process, biological regulation, cellular component organization or biogenesis, and localization. The protein-containing complex, organelle, and cell were most significant among the cellular components (CC). Enriched molecular function (MF) terms included transcription regulator activity, binding, structural molecule activity, catalytic activity, and transporter activity. A detailed GO-Slim classification within three major functional categories is shown in Figures 4(a)–4(c). The top 1 enriched GO-Slim terms within three major functional categories are shown in Figures 4(d)–4(f). The results showed that the metabolic process was dominant in the BP terms, the organelle in the CC terms, and catalytic activity in the MF terms. 75.86% of the metabolic process-related genes were involved in the organic substance metabolic process. The genes related to the organelle mainly participated in the intracellular organelle. The term of catalytic activity mainly included ligase activity, oxidoreductase activity, transferase activity, catalytic activity, hydrolase activity, and lyase activity. GO-Slim classification revealed that most of high-confidence miRNA targets were related to a series of basic biological processes, even under the heat conditions. In addition to basic life processes, targets were also associated with response to stimulus and oxidoreductase activity which were closely related to the heat stress.

### 3.5. Functional Identification of High-Confidence Target Genes

OGRO on the Q-TARO website (http://qtaro.abr.affrc.go.jp/ogro), which is an online database of functionally characterized genes in rice, collects nearly 1100 genes. A search of the 178 high-confidence target genes in the OGRO database identified 13 functionally characterized genes (Table 2), and most of these characterized high-confidence target genes were related to stress tolerance, sterility, panicle flower, and flowering, which suggested that the important roles of the high-confidence miRNA targets were identified in the heat-treated rice post-meiosis panicle.

There were 9 high-confidence target genes associated with resistance or tolerance, and most of them were downregulated. It was remarkable that the genes for LOC_Os05g51830 (*OsHDT1*), LOC_Os05g30220 (*OsRP1L1*), LOC_Os05g47780 (*OsHRZ2*), LOC_Os06g46270 (*OMTN4*), LOC_Os10g38060 (*OsPLDβ1*), and LOC_Os10g40100 (*OsRLCK306*) were continuously downregulated by the heat stress. At the same time, LOC_Os01g64980 (*Os-pollux*), LOC_Os08g45000 (*OsPT6*), and LOC_Os12g18360 (*Pi-ta*) were upregulated during the heat treatment. *OsHDT1*-silencing or *OsPLDβ1*-knockdown plants showed the accumulation of reactive oxygen species (ROS) [24, 25]. *OsHDT1* and *OsPLDβ1*, as high-confidence target genes of osa-miR414 and osa-miR319, respectively, were significantly downregulated under the heat stress. These showed that osa-miR414 and osa-miR319 might increase the accumulation of ROS to resist heat stress by downregulating the expression of *OsHDT1* and *OsPLDβ1*. Suppressing the expression of *OsRLCK306* (NRRB) could enhance the resistance to the bacterial leaf streak in rice [26]. The *Pi-ta* gene in rice could be used to control rice blast disease caused by *Magnaporthe oryzae* [27]. The increased disease resistance was accompanied by the accumulation of salicylic acid (SA) and by the induced expression of a number of defense-responsive genes [26]. SA could alleviate the damage of floret differentiation caused by the heat stress at the floret differentiation stage and prevent the spikelet degeneration caused by the heat stress [28, 29]. *OsRLCK306* and *Pi-ta*, as a high-confidence target gene of osa-miR166 and osa-miR395, respectively, showed significant expression changes under the heat stress. It means that osa-miR166 and osa-miR395 might regulate the content of SA in plants by regulating the expression of *OsRLCK306* and *Pi-ta* to resist the heat stress. *OsRP1L1* was involved in responses to several plant growth regulators and environmental stresses [30]. *OsHRZ2*, as an iron-binding hemerythrin RING ubiquitin ligase, could regulate the iron acquisition. Overexpression of *OMTN4* negatively regulated drought resistance in rice at the reproductive stage [18]. *Os-pollux*, as a cation channel protein, was expressed in roots, leaves, stems, and panicles [31]. *OsPT6* was related to the sugar transporter family. All these five significant expression changes of stress-related genes showed that multiple functions of the identified candidate genes were very important in response to the heat stress and the complexity of heat response in rice panicles. These nine high-confidence target genes were following high-confidence target pairs of miRNAs: osa-miR164, osa-miR166, osa-miR169, osa-miR319, osa-miR390, osa-miR395, and osa-miR399, which suggested the important roles of the miRNAs identified in the heat-treated rice post-meiosis panicle.

In this work, sterility-related gene tapetum degeneration retardation gene (LOC_Os02g18080, *TDR*), as a high-confidence target gene of osa-miR437, showed significant upregulation under the heat stress in the rice post-meiosis panicle. However, under normal growth conditions, *TDR* remained at a lower expression level in various organs at different developmental stages in rice except for the flag leaves and roots after flowering [32]. This significant expression change of *TDR* suggested the great importance of osa-miR437 in response to the heat stress in the post-meiosis panicle.

There were 4 high-confidence target genes related to the panicle flower, which included LOC_Os02g12380 (*HDA710*), LOC_Os04g55560 (*SHAT1*), OMTN4, and LOC_Os04g46580 (*OsSPL7*). Previous research showed that inactivation of *HDA710* by RNAi affected vegetative growth, while downregulation of a closely related homolog *HDA703* by amiRNA reduced rice peduncle elongation and fertility [33]. It suggested the importance of LOC_Os02g12380 in anther development under the heat stress. *SHAT1*, as a target of osa-miR172, was significantly upregulated under the heat stress. *SHAT1* is a member of the AP2 family of genes. miRNA172-mediated cleavage of SHAT1 might be essential for normal flower opening in rice [17]. Arabidopsis AP2 was reported to participate in a self-feedback loop through negatively regulating its inhibitor miR172 [34]. It means that osa-miR172 and its target gene *SHAT1* might play important roles in protecting the rice panicle under the heat stress. *OMTN4*, which is the target of osa-miR164 and positive regulator of heading and senescence during the reproductive phase, was responsive to abiotic stresses. Notably, *OMTN4* exhibited particularly high levels of expression in stamen in rice ZH11 grown under normal growth conditions [18]. In our study, *OMTN4* were downregulated in the rice post-meiosis panicle. This means that osa-miR164 might negatively affect rice heading and senescence during the reproductive phase under the heat stress. *OsSPL7* expressed in young panicles and might be involved in panicle development [35]. In our study, both miR156 and miR529 were downregulated and their common target *OsSPL7* was upregulated under the heat stress in the rice post-meiosis panicle. These might be disadvantageous to spikelet development because downregulation of osa-miR156 and overexpression *OsSPL7* would lead to decreased tillers, smaller panicle, lower fertility, and eventually reduced rice yield [15, 35].

Increased expression of *OsHDT1* could suppress the overdominant expression of flowering time repressors in the hybrid leading to early flowering under the long day condition [36]. In our study, flowering-related *OsHDT1*, as a high-confidence target gene of osa-miR414, was significantly downregulated under the heat stress. This means that the inhibition of *OsHDT1* on the flowering suppressor gene is weak, so reducing the expression of the flowering gene and delaying the heading stage of rice are more conducive to improve the heat tolerance of rice under the short time heat stress.

### 3.6. Construction of miRNA-PPI Interaction Network

**Figure 5:**
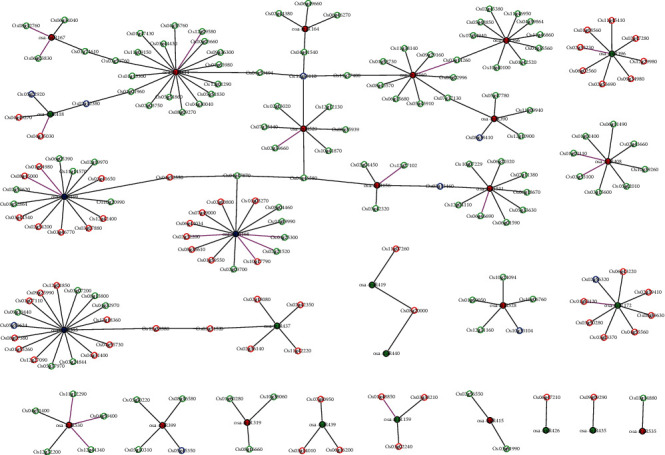
Network visualization of miRNA-PPI in rice post-meiosis panicles. The node colors of red, green, and blue denote three expression patterns: upregulated, downregulated, and up-/downregulated pattern, respectively. The black edges represent cleavage, and the red edges represent translation inhibition in the miRNA-target module. The edge color of PPI denotes the combined score. The larger the combined score is, the darker the color is. The “LOC” of the prefix for the gene name in the network was omitted for convenience.

The network visualization of miRNA-PPI is shown in Figure 5. From Figure 5, we could draw the following conclusions: (1) Most of the target genes were regulated through cleavage rather than through translation inhibition. (2) The maximum number of targets was 18 for osa-miR414. (3) Most of miRNA families regulated multiple genes, and a small number of genes could be in turn regulated by multiple miRNA families. These results indicated that there was a degree of cooperation among the miRNA families under the heat stress in rice. (4) Three miRNA coregulatory modules, i.e., groups of miRNAs that regulated groups of target genes in concert, were revealed. Among them, for the osa-miR529-centered coregulatory module, osa-miR529 might coexpress with 11 miRNAs through the target genes and coregulate 102 high-confidence downstream targets. Previous studies showed that miR529 might regulate the panicle development in rice and the overexpression of miR529a dramatically affected the panicle architecture [16]. The overexpression of osa-miR156 resulted in strongly reduced panicle size and delayed flowering. These showed that osa-miR156 and *OsSPL* target genes were involved in the flower development of rice [35]. miR167 and its family members could affect the pollen development [37]. The low level of *OsARF12* maintained by osa-miR167 during anther development was essential for the development and maturation of pollen grains [38]. miR166 and its target *PHB* regulated SPL/NZZ, which controlled microsporogenesis [39]. The functions of miR160 and miR164 were also related to the flower development in the PNRD database [40]. Therefore, we speculated that the coregulatory module with miR529 as the hub might play an important role in the development of the panicle and flower. miR395 and miR437 could coexpress through the interaction between their target proteins. miRNA419 was coexpressed with miR440 through their common target gene. One mRNA, LOC_Os04g46580 (*OsSPL7*), was a common target of two significantly upregulated miRNAs, miR156 and miR529. The coregulation of miR529 and miR156 was expected, since miR529 was evolutionarily related to miR156 and showed 14-16 nucleotide similarities with miR156 [41, 42]. All this information indicated that the multiple interactions existed not only in the miRNAs and target genes but also between miRNA-target proteins.

Previous work demonstrated that coordinate miRNA regulation existed in Drosophila and mammals [43, 44]. A recent report found that a similar mechanism existed in rice under the drought stress [45]. In our study, three miRNA coregulatory modules suggested that there was also a coordinate miRNA regulation in the rice post-meiosis panicle under the heat stress. Through synergism, miRNAs may enhance repression effects and reinforce their regulatory efficacy. Moreover, synergistic gene regulation was less dependent on miRNA abundance, so limited miRNA could regulate more genes. The identification of coregulatory miRNA modules would help researchers understand the mechanisms of miRNA involvement in the heat stress tolerance in the rice post-meiosis panicle.

## 4. Conclusions

In conclusion, our genome-wide microarray analysis identified a set of high-confidence miRNA target pairs in rice panicles at the post-meiosis stage. The expression pattern analysis of high-confidence miRNA target pairs showed that most miRNAs resisted the heat stress by downregulating their target genes and a time lag existed between miRNA expression and their target gene expression. PANTHER GO-Slim analysis showed that these high-confidence miRNA target genes were mainly involved in a series of basic biological processes. In addition, targets were also involved in response to stimulus and oxidoreductase activity which were closely related to the heat stress. The query of the OGRO database identified 13 genes that were functionally characterized. The functional characters of these 13 genes showed that osa-miR164, osa-miR166, osa-miR169, osa-miR319, osa-miR390, osa-miR395, and osa-miR399 might play important roles in heat resistance; osa-miR172 might play important roles in protecting the rice panicle under heat stress. However, osa-miR437, osa-miR418, osa-miR164, osa-miR156, and osa-miR529 might negatively affect rice fertility and panicle flower; osa-miR414 might inhibit flowering gene expression by downregulation of LOC_Os 05g51830 to delay the heading of rice. The visualization of the miRNA-PPI network supported the existence of a coregulatory network triggered by the heat stress. Further verification of these hub miRNAs and genes as coregulatory nodes might be helpful to develop novel molecular markers for breeding new rice varieties with enhanced heat tolerance. In-depth analysis of coregulatory networks may help better understand the regulatory mechanism of miRNAs under the heat stress.

## Figures and Tables

**Table 1:** High-confidence miRNA target pairs in rice post-meiosis panicle under the heat stress.

DE-miRNA families		Target genes	
osa-miR156	↑	LOC_Os02g04450, LOC_Os03g42320, LOC_Os04g46580, LOC_Os12g27102	↓
		LOC_Os03g13460	↑↓
osa-miR159	↓	LOC_Os01g48850, LOC_Os03g02240, LOC_Os03g38210	↑
osa-miR160	↑	LOC_Os01g58730, LOC_Os03g14260, LOC_Os05g43910, LOC_Os06g15680, LOC_Os07g37130, LOC_Os08g02996, LOC_Os08g43570, LOC_Os09g29160, LOC_Os11g27400, LOC_Os11g38140	↓
osa-miR164	↑	LOC_Os02g44380, LOC_Os04g41540, LOC_Os06g46270, LOC_Os06g49660	↓
osa-miR166	↑	LOC_Os01g42520, LOC_Os02g45380, LOC_Os03g14260, LOC_Os04g35560, LOC_Os04g39864, LOC_Os05g48850, LOC_Os07g34940, LOC_Os10g40100, LOC_Os11g46860, LOC_Os11g46950	↓
osa-miR167	↑	LOC_Os01g74610, LOC_Os06g03830, LOC_Os06g34040, LOC_Os08g12760	↓
osa-miR168	↑↓	LOC_Os01g19990, LOC_Os01g25300, LOC_Os02g03700, LOC_Os02g21520, LOC_Os04g47870, LOC_Os08g04460	↓
		LOC_Os01g03270, LOC_Os01g59550, LOC_Os02g10800, LOC_Os02g32200, LOC_Os06g44034, LOC_Os07g49000, LOC_Os08g38610, LOC_Os10g17790	↑
osa-miR169	↑↓	LOC_Os01g52864, LOC_Os02g19970, LOC_Os02g53620, LOC_Os06g05390, LOC_Os10g20990, LOC_Os11g14570	↓
		LOC_Os01g41650, LOC_Os01g64980, LOC_Os02g54200, LOC_Os03g07880, LOC_Os03g44540, LOC_Os03g46770, LOC_Os04g52550, LOC_Os08g45000, LOC_Os12g42400	↑
osa-miR172	↓	LOC_Os02g56320	↑↓
		LOC_Os01g52120, LOC_Os02g39410, LOC_Os03g13370, LOC_Os03g50280, LOC_Os04g55560, LOC_Os06g43220, LOC_Os08g39630	↑
osa-miR319	↑	LOC_Os01g60280, LOC_Os08g16660, LOC_Os10g38060	↓
osa-miR390	↑	LOC_Os05g47780, LOC_Os07g37130, LOC_Os11g09940, LOC_Os12g10900	↓
		LOC_Os08g38410	↑↓
osa-miR395	↑↓	LOC_Os03g07200, LOC_Os03g24844, LOC_Os04g32970, LOC_Os05g37970, LOC_Os08g13800, LOC_Os09g39440	↓
		LOC_Os05g50624	↑↓
		LOC_Os01g07110, LOC_Os01g55260, LOC_Os04g31400, LOC_Os04g55730, LOC_Os08g07380, LOC_Os09g25990, LOC_Os12g01850, LOC_Os12g18360, LOC_Os12g27090, LOC_Os12g38380	↑
osa-miR396	↓	LOC_Os01g08560, LOC_Os02g33230, LOC_Os02g47280, LOC_Os02g53690, LOC_Os06g02560, LOC_Os09g34980, LOC_Os11g45410, LOC_Os12g29980	↑
osa-miR399	↑	LOC_Os05g10310, LOC_Os05g30220, LOC_Os08g16580	↓
		LOC_Os05g45350	↑↓
osa-miR408	↑	LOC_Os01g01400, LOC_Os01g02110, LOC_Os02g43660, LOC_Os03g15600, LOC_Os03g53100, LOC_Os05g42010, LOC_Os06g11490, LOC_Os10g39260	↓
osa-miR414	↑	LOC_Os01g01960, LOC_Os01g13300, LOC_Os01g47430, LOC_Os01g63980, LOC_Os02g03750, LOC_Os03g44430, LOC_Os03g59760, LOC_Os04g30040, LOC_Os04g35760, LOC_Os04g59494, LOC_Os05g51830, LOC_Os05g51860, LOC_Os08g09270, LOC_Os09g36300, LOC_Os10g03660, LOC_Os11g09150, LOC_Os12g01290, LOC_Os12g09580	↓
osa-miR415	↑	LOC_Os02g56550, LOC_Os03g61990	↓
osa-miR418	↓	LOC_Os02g12380, LOC_Os05g22920	↑↓
		LOC_Os04g35030, LOC_Os04g49270	↑
osa-miR419	↓	LOC_Os08g20000, LOC_Os11g07260	↑
osa-miR426	↓	LOC_Os06g47210	↑
osa-miR435	↓	LOC_Os09g09290	↑
osa-miR437	↓	LOC_Os02g18080, LOC_Os02g36140, LOC_Os02g42350, LOC_Os03g51520, LOC_Os11g42220	↑
osa-miR439	↓	LOC_Os03g14010, LOC_Os06g16200, LOC_Os07g30950	↑
osa-miR440	↓	LOC_Os08g20000	↑
osa-miR528	↑	LOC_Os01g69050, LOC_Os10g06760, LOC_Os10g24094, LOC_Os12g31160	↓
		LOC_Os10g33104	↑↓
osa-miR529	↑	LOC_Os02g13020, LOC_Os02g39660, LOC_Os04g46580, LOC_Os07g35140, LOC_Os08g35939, LOC_Os10g41870, LOC_Os12g12130	↓
		LOC_Os12g38110	↑↓
osa-miR530	↑	LOC_Os04g51400, LOC_Os04g59400, LOC_Os11g12290, LOC_Os12g32200, LOC_Os12g44340	↓
osa-miR531	↑	LOC_Os05g33630, LOC_Os06g01590, LOC_Os06g18670, LOC_Os06g20320, LOC_Os06g46690, LOC_Os07g11380, LOC_Os10g07229, LOC_Os12g04110	↓
		LOC_Os03g13460	↑↓
osa-miR535	↑	LOC_Os03g14880	↓

Note: ↑, ↓, and ↑↓ indicate upregulated, downregulated, and up-/downregulated types in rice post-meiosis panicle under the heat stress, respectively.

**Table 2:** The characterization of high-confidence target genes in the OGRO database.

Locus_id	Gene_symbol	Character_major	Character_minor	Isolation	Objective	Reference (doi)
LOC_Os05g51830	OsHDT1	Resistance or tolerance	Bacterial blight resistance	Knockdown overexpression	Resistance to *Magnaporthe oryzae* and *Xanthomonas oryzae pv. oryzae*	10.1105/tpc.112.101972
LOC_Os05g51830	OsHDT1	Resistance or tolerance	Blast resistance	Knockdown overexpression	Resistance to *Magnaporthe oryzae* and *Xanthomonas oryzae pv. oryzae*	10.1105/tpc.112.101972
LOC_Os01g64980	Os-pollux	Resistance or tolerance	Other soil stress tolerance	Mutant	Mycorrhizal symbiosis	10.1104/pp.108.131540
LOC_Os05g30220	OsRP1L1	Resistance or tolerance	Bacterial blight resistance	Overexpression	Resistance to *Xanthomonas oryzae pv. oryzicola*	10.1007/s11033-011-1122-6 10.1007-s11105-012-0537-0
LOC_Os05g47780	OsHRZ2	Resistance or tolerance	Other soil stress tolerance	Knockdown	Fe acquisition	10.1038/ncomms3792
LOC_Os06g46270	OMTN4	Resistance or tolerance	Drought tolerance	Overexpression	Drought sensitivity	10.1093/jxb/eru072
LOC_Os08g45000	OsPT6	Resistance or tolerance	Other soil stress tolerance	Knockdown	Phosphate uptake and translocation	10.1111/j.1365-313X.2008.03726.x
LOC_Os10g38060	OsPLD*β*1	Resistance or tolerance	Blast resistance	Knockdown	Resistance to *Pyricularia grisea* and *Xanthomonas oryzae pv. oryzae*	10.1104/pp.108.131979
LOC_Os10g40100	OsRLCK306	Resistance or tolerance	Blast resistance	Knockdown	Promote invasion of bacteria (Xoc)	10.1007/s11033-014-3069-x
LOC_Os12g18360	Pi-ta	Resistance or tolerance	Blast resistance	Natural variation	Resistance to *Magnaporthe grisea*	10.1105/tpc.12.11.2033
LOC_Os02g18080	TDR	Morphological trait	Sterility	Others	“Delated/non-degradation of tapetum tissue, collapse of the haploid microspores”	10.1007/s11032-013-9972-3
LOC_Os04g46580	OsSPL7	Morphological trait	Panicle flower	Overexpression	Panicle development	DOI:10.1104/pp.106.084475
LOC_Os02g12380	HDA710	Morphological trait	Panicle flower	Knockdown	Flag leaf morphology, semidwarf, awn development	10.1016/j.bbrc.2009.07.162
LOC_Os04g55560	SHAT1	Morphological trait	Panicle flower	Mutant	Seed shattering, floral organ identity	10.1105/tpc.111.094383
LOC_Os06g46270	OMTN4	Physiological trait	Panicle flower	Overexpression	Low spikelet fertility	10.1093/jxb/eru072
LOC_Os05g51830	OsHDT1	Physiological trait	Flowering	Overexpression	Flowering time in hybrid, chromatin modification	10.1371/journal.pone.0021789
LOC_Os02g12380	HDA710	Morphological trait	Culm leaf	Knockdown	Flag leaf morphology, semidwarf, awn development	10.1016/j.bbrc.2009.07.162
LOC_Os06g46270	OMTN4	Morphological trait	Culm leaf	Knockdown overexpression	“Leaf rolling, wilting during drought stress”	10.1093/jxb/eru072
LOC_Os02g12380	HDA710	Morphological trait	Dwarf	Knockdown	Flag leaf morphology, semidwarf, awn development	10.1016/j.bbrc.2009.07.162
LOC_Os10g38060	OsPLD*β*1	Physiological trait	Germination dormancy	Knockdown	Sensitivity to ABA during germination stage	10.1038/cr.2007.77

## Data Availability

All research data used to support the findings of this study are available from the corresponding author upon request.

## Supplementary Materials

Supplementary Materials Table S1: the normalized data of expressed miRNAs and mRNAs at five time points. Table S1 contains the results of the normalized data of expressed miRNAs and mRNAs at five time points. The microarray data are shown on the left side, and the results of the differential expression analysis are shown on the right side. Table S2-1: the list of 124 differentially expressed miRNAs in rice post-meiosis panicle. Table S2-1 contains the list of 124 DE-miRNAs in rice post-meiosis panicle under the heat stress. Eighty-six of 124 DE-miRNAs are upregulated and 38 of them are downregulated. Table S2-2: the mature sequences of 124 differentially expressed miRNAs. Table S2-2 contains 154 mature sequences of 124 DE-miRNAs. Table S3-1: putative target genes of 37 DE-miRNA families. Table S3-1 contains 1289 putative target genes of 37 DE-miRNA families. Table S3-2: the expression analysis of target genes under the heat stress in rice post-meiosis panicle. Table S3-2 contains the expression analysis results of 838 of 1289 putative target genes. Table S3-3 differentially expressed target genes of 37 DE-miRNA families under the heat stress in rice post-meiosis panicle. Table S3-3 contains the identified 351 DEGs of 37 DE-miRNA families under the heat stress in rice post-meiosis panicle. Table S4: the list of 178 high-confidence miRNA target genes' description. Table S4 contains the list of 178 high-confidence miRNA target genes' description.
